# A conifer genomics resource of 200,000 spruce (*Picea *spp.) ESTs and 6,464 high-quality, sequence-finished full-length cDNAs for Sitka spruce (*Picea sitchensis*)

**DOI:** 10.1186/1471-2164-9-484

**Published:** 2008-10-14

**Authors:** Steven G Ralph, Hye Jung E Chun, Natalia Kolosova, Dawn Cooper, Claire Oddy, Carol E Ritland, Robert Kirkpatrick, Richard Moore, Sarah Barber, Robert A Holt, Steven JM Jones, Marco A Marra, Carl J Douglas, Kermit Ritland, Jörg Bohlmann

**Affiliations:** 1 Michael Smith Laboratories, University of British Columbia, Vancouver, British Columbia, V6T 1Z4, Canada; 2 British Columbia Cancer Agency Genome Sciences Centre, Vancouver, British Columbia, V5Z 4E6, Canada; 3 Department of Botany, University of British Columbia, Vancouver, British Columbia, V6T 1Z4, Canada; 4 Department of Forest Sciences, University of British Columbia, Vancouver, British Columbia, V6T 1Z4, Canada; 5 Department of Biology, University of North Dakota, Grand Forks, ND, 58202-9019, USA

## Abstract

**Background:**

Members of the pine family (Pinaceae), especially species of spruce (*Picea *spp.) and pine (*Pinus *spp.), dominate many of the world's temperate and boreal forests. These conifer forests are of critical importance for global ecosystem stability and biodiversity. They also provide the majority of the world's wood and fiber supply and serve as a renewable resource for other industrial biomaterials. In contrast to angiosperms, functional and comparative genomics research on conifers, or other gymnosperms, is limited by the lack of a relevant reference genome sequence. Sequence-finished full-length (FL)cDNAs and large collections of expressed sequence tags (ESTs) are essential for gene discovery, functional genomics, and for future efforts of conifer genome annotation.

**Results:**

As part of a conifer genomics program to characterize defense against insects and adaptation to local environments, and to discover genes for the production of biomaterials, we developed 20 standard, normalized or full-length enriched cDNA libraries from Sitka spruce (*P. sitchensis*), white spruce (*P. glauca*), and interior spruce (*P. glauca-engelmannii *complex). We sequenced and analyzed 206,875 3'- or 5'-end ESTs from these libraries, and developed a resource of 6,464 high-quality sequence-finished FLcDNAs from Sitka spruce. Clustering and assembly of 147,146 3'-end ESTs resulted in 19,941 contigs and 26,804 singletons, representing 46,745 putative unique transcripts (PUTs). The 6,464 FLcDNAs were all obtained from a single Sitka spruce genotype and represent 5,718 PUTs.

**Conclusion:**

This paper provides detailed annotation and quality assessment of a large EST and FLcDNA resource for spruce. The 6,464 Sitka spruce FLcDNAs represent the third largest sequence-verified FLcDNA resource for any plant species, behind only rice (*Oryza sativa*) and Arabidopsis (*Arabidopsis thaliana*), and the only substantial FLcDNA resource for a gymnosperm. Our emphasis on capturing FLcDNAs and ESTs from cDNA libraries representing herbivore-, wound- or elicitor-treated induced spruce tissues, along with incorporating normalization to capture rare transcripts, resulted in a rich resource for functional genomics and proteomics studies. Sequence comparisons against five plant genomes and the non-redundant GenBank protein database revealed that a substantial number of spruce transcripts have no obvious similarity to known angiosperm gene sequences. Opportunities for future applications of the sequence and clone resources for comparative and functional genomics are discussed.

## Background

Conifers (members of the pine family) have very large genomes (10 to 40 Gb, [1]), and this poses difficulties for both structural and functional genomic studies. In addition, their generation times are long and their habitual out-breeding nature prevents the development of inbred strains useful for genetics research. A further difficulty in conifer genomics is the large evolutionary distance between conifers and angiosperms (i.e., flowering plants), separated by 300 million years of evolution [2], which severely restricts gene comparisons of conifers with angiosperms. While there are several completely sequenced angiosperm genomes, as well as high-quality sequence-finished full-length (FL)cDNA resources, for Arabidopsis [3,4], rice [5-7], poplar (*Populus trichocarpa*; [8,9]), grapevine (*Vitis vinifera*; [10]), and a moss (*Physcomitrella patens*; [11]), these basic genomics resources have not yet been developed for the conifer phyla or for any other gymnosperm.

In species with large genomes, a critical first step for genome characterization is to survey the expressed genes. A common approach to characterize the expressed genome is to sequence cDNA libraries and to assemble large collections of expressed sequence tags (ESTs) [12]. In the absence of a conifer genome sequence, large and deep EST collections are particularly useful. Sequencing of cDNA libraries constructed from diverse tissues and developmental stages, and from materials subjected to diverse environmental conditions or treatments, enhances the diversity of genes captured in EST populations. In addition, normalization techniques reduce the frequency of highly expressed genes and increase the rate of rare gene discovery [13,14], thus providing more comprehensive coverage of the expressed genome.

In conifers, gene discovery via EST sequencing was first conducted in loblolly pine (*Pinus taeda*; [15]), the most economically important tree species in the southeastern USA. The early emphasis in loblolly pine was on wood forming tissues [16], but newer projects have involved treatments such as drought stress [17] and embryogenesis [18]. As of May 2008, the loblolly pine EST collection contains more than 328,000 sequences [19]. Recent EST projects with species of spruce have used tissues related to shoot growth and xylem development in white spruce [20,21], wound treatment in interior spruce [21], root development in Sitka spruce [21], and xylem development and bud burst in Norway spruce (*P. abies*; [22,23]). EST resources have also been developed for a few other gymnosperm species outside of the pine family, such as cycas (*Cycas rumphii*; [24]), ginkgo (*Ginkgo biloba*; [25]), Japanese yew (*Taxus cuspidata*; [26]), Japanese cedar (*Cryptomeria japonica*; [27,28]) and Hinoki cypress (*Chamaecyparis obtusa*; [28]).

In addition to deep EST sampling, other important components of a cDNA sequence resource are the quality and length of sequence coverage for a given gene. Ideally, FLcDNA clones that capture the entire mature transcript of a gene should be identified and completely sequenced with high accuracy. FLcDNA sequences should span not only the protein-coding open reading frame (ORF) region but also the non-coding 5' and 3' untranslated regions (UTRs). Most importantly, true FLcDNA sequences should be derived from a single individual FLcDNA clone. Using individual clones prevents the assembly of chimeric FLcDNA sequences consisting of ESTs from multiple cDNA clones representing closely related genes. Furthermore, allelic nucleotide polymorphisms and alternatively spliced variants of a gene are difficult to detect using *in silico *assembled sequence contigs from multiple clones. To further discriminate among closely related genes, the authenticity of sequences should be verified by re-sequencing of the same clone (sequence verification). Compared to single-pass ESTs or *in silico *assembled sequence contigs originating from multiple clones, sequence-verified FLcDNA clones offer several advantages for comparative, structural, and functional genome analyses, in particular for conifers with their great evolutionary distance from angiosperms. First, the complete protein-coding regions of FLcDNAs can be unambiguously identified. An accurate prediction of full-length protein sequences aides in the correct identification of distant angiosperm homologues. Second, in anticipation of a future conifer genome sequence, FLcDNAs can be used to improve gene prediction from genomic sequences as demonstrated in Arabidopsis [29-31] and poplar [8,9]. Third, FLcDNA clones can be used for functional characterization of conifer genes using biochemical approaches [e.g., [32,33]] or for functional complementation of mutants in heterologous systems. Given the lack of knock-out mutants in conifers and the slow process of generating knock-down mutants in conifers, biochemical approaches and heterologous complementation that rely on FLcDNA clones are essential tools for functional genomics in conifers. Finally, FLcDNAs can be used to accurately identify peptides in large-scale conifer proteome analyses [34,35].

Despite their immense value, sequence-verified FLcDNA clones have not been generated in most plant species subjected to genome analysis. Only a few resources of large and sequence-verified FLcDNA data sets have been generated for angiosperm plant species; namely, for Arabidopsis [4], rice [7], and poplar [9]. In contrast, no substantial FLcDNA resource has been reported for a conifer or any other gymnosperm species. The Conifer Forest Health genomics project "Treenomix" [36] aims to develop genomic resources for spruce, characterize mechanisms of resistance against insect pests and adaptation to local environments, and identify genes for the formation of oleoresin-based terpenoid biomaterials [37-43]. Here, we report on a comprehensive spruce EST and FLcDNA resource and discuss its utility for conifer genomics. A total of 206,875 ESTs were obtained by sequencing 20 standard, normalized or full-length cDNA libraries derived from Sitka spruce, white spruce, and interior spruce. Analysis of ESTs identified 46,745 putative unique transcripts (PUTs). We describe advantages covered by the first large set of 6,464 sequence-verified, high-quality FLcDNAs obtained from a single clonally propagated tree of Sitka spruce.

## Results

### Sequencing and assembly of spruce ESTs

We constructed 20 unidirectional standard, normalized or full-length enriched cDNA libraries from various tissues, developmental stages, and stress treatments of Sitka spruce, white spruce and interior spruce (Table 1). Several libraries were made from trees subjected to insect feeding by white pine weevils (*Pissodes strobi*) or spruce budworms (*Choristoneura occidentalis*), or to herbivory-simulation treatments such as mechanical wounding or methyl jasmonate application. From these libraries, we obtained 206,875 EST sequences, consisting of 165,403 3'-end EST sequences and 41,472 5'-end EST sequences (Table 2). We initially focused on 3'-end sequencing. Subsequent sequence reads from 5'-ends were performed as paired end reads, primarily from clones derived from FLcDNA libraries, to support the identification of a non-redundant FLcDNA set for complete insert sequencing. Removing low-quality and vector sequences (see Table 2 for criteria), as well as any obvious contaminant sequences, provided a database containing 147,146 high-quality (hq) 3' ESTs (88.9% success rate) with an average read length of 656 bp (Table 2). When we analyzed the 147,146 hq 3'-end ESTs using the CAP3 program ([44]; assembly criteria: 95% identity, 40 bp window), 120,342 ESTs assembled into 19,941 contigs and the remaining 26,804 ESTs were classified as singletons, suggesting a combined total of 46,745 PUTs across Sitka spruce, white spruce and interior spruce (Table 2). On average, contigs contained six assembled EST sequences. Only 88 contigs consisted of greater than 50 ESTs. The five largest contigs contain 618 (aspartyl protease), 229 (ribulose biphosphate carboxylase small subunit), 222 (metallothionein), 209 (translationally controlled tumor protein) and 172 (no significant match) ESTs. The proportion of EST sequences from organelles was small. Known and putative mitochondrial and chloroplast sequences contribute only 285 (0.19%) and 787 (0.53%) ESTs to the entire data set, respectively. In separate species-specific assemblies using ESTs from only white spruce or Sitka spruce, we identified 23,963 PUTs (72,649 3'-end EST sequences, 10,948 contigs and 13,015 singletons) and 17,988 PUTs (49,198 3'-end EST sequences, 6,918 contigs and 11,070 singletons), respectively.

**Table 1:** Libraries, tissue sources and spruce species for sequences described in this study

cDNA Library	Tissue/Developmental stage	Species (genotype)
WS-ES-A-1^a^	Young shoots harvested from 25-year old trees^d^.	*P. glauca *(PG-29)
WS-PS-A-2^a^	Flushing buds, young shoots and mature shoots harvested from 25-year old trees^d^.	*P. glauca *(PG-29)
WS-X-A-3^a^	Early (June 15^th^), mid (July 10^th^) and late (August 17^th^) season outer xylem harvested from 25-year old trees^d^.	*P. glauca *(PG-29)
IS-B-A-4^a^	Bark tissue (with phloem and cambium) harvested after razor blade wounding and treatment with 0.01% methyl jasmonate. Tissue was collected 0 (untreated), 3, 6 and 12 h post-treatment^e^.	*P. glauca *× *P. engelmannii *(Fal-1028)
SS-R-A-5^a^	Young growth (terminal 1–3 cm) and mature growth (distal to terminal 1–3 cm) roots^e^.	*P. sitchensis *(Gb2-229)
WS-PP-A-6^a^	Early (June 15^th^), mid (July 10^th^) and late (August 17^th^) season phloem harvested from 25-year old trees^d^.	*P. glauca *(PG-29)
IS-B-A-7^a^	Bark tissue (with phloem and cambium) harvested after razor blade wounding and treatment with 0.01% methyl jasmonate. Tissue was collected 24 h, 2 d, 4 d and 8 d post-treatment^e^.	*P. glauca *× *P. engelmannii *(Fal-1028)
WS-PS-N-A-8^b^	Flushing buds, young shoots and mature shoots harvested from 25-year old trees^d^.	*P. glauca *(PG-29)
WS-X-N-A-9^b^	Early (June 15^th^), mid (July 10^th^) and late (August 17^th^) season outer xylem harvested from 25-year old trees^d^.	*P. glauca *(PG-29)
IS-B-N-A-10^b^	Bark tissue (with phloem and cambium attached) harvested after razor blade wounding and treatment with 0.01% methyl jasmonate. Tissue was collected 0 h (untreated), 3 h, 6 h, 12 h, 24 h, 2 d, 4 d and 8 d post-treatment^e^.	*P. glauca *× *P. engelmannii *(Fal-1028)
SS-R-N-A-11^b^	Young growth (terminal 1–3 cm) and mature growth (distal to terminal 1–3 cm) roots^e^.	*P. sitchensis *(Gb2-229)
WS-PP-N-A-12^b^	Early (June 15^th^), mid (July 10^th^) and late (August 17^th^) season phloem harvested from 25-year old trees^d^.	*P. glauca *(PG-29)
SS-IB-A-FL-13^c^	Bark tissue (with phloem and cambium attached) harvested after continuous feeding by *Pissodes strobi *weevils. Tissue was collected 2, 6 and 48 h post-treatment^e^.	*P. sitchensis *(FB3-425)
SS-IL-A-FL-14^c^	Green portion of leader tissue harvested after continuous feeding by *Choristoneura occidentalis *budworms. Tissue was collected 3 h, 6 h, 12 h, 24 h, 52 h, 4 d, 6 d, 8 d and 10 d post-treatment^e^.	*P. sitchensis *(FB3-425)
SS-IB-A-FL-15^c^	Bark tissue (with phloem and cambium attached) harvested after continuous feeding by *P. strobi *weevils. Tissue was collected 2, 6 and 48 h post-treatment^e^.	*P. sitchensis *(FB3-425)
WS-SE-A-16^a^	Somatic embryo tissue harvested at the callus stage, and after 2, 4 and 6 weeks of growth on media supplemented with abscisic acid and indole-3-butyric acid.	*P. glauca *(I-1026)
WS-MC-A-17^a^	Cones harvested from 25-year old trees^d^	*P. glauca *(11)
WS-SE-N-A-18^b^	Somatic embryo tissue harvested at the callus stage, and after 2, 4 and 6 weeks of growth on media supplemented with abscisic acid and indole-3-butyric acid.	*P. glauca *(I-1026)
WS-SE-N-A-19^b^	Somatic embryo tissue harvested at the callus stage, and after 2, 4 and 6 weeks of growth on media supplemented with abscisic acid and indole-3-butyric acid.	*P. glauca *(I-1026)
WS-MC-N-A-20^b^	Cones harvested from 25-year old trees^d^	*P. glauca *(11)

^a^Standard cDNA library; ^b^Normalized cDNA library; ^c^Full-length cDNA library; ^d^Field site located at Kalamalka Research Station in Vernon, British Columbia; ^e^One- or two-year old trees grown in potted soil under greenhouse conditions at the University of British Columbia

**Table 2:** Spruce EST summary

Total sequences	206,875
Number of 5' sequences	41,472
Number of 3' sequences	165,403
Average assembled 3' EST length (bp)^a^	656.4
Number of high-quality 3' sequences^b^	147,146
Number of contigs^c^	19,941
Number of singletons	26,804
Number of putative unique transcripts^d^	46,745
Number of assembled 3' ESTs with^e^	
Significant BLASTX match	96,454
No significant BLASTX match	50,692
Average number of contig members	6.03
Number of contigs containing	
2 ESTs	6,050
3–5 ESTs	7,449
6–10 ESTs	3,841
11–20 ESTs	1,941
21–50 ESTs	572
>50 ESTs	88

^a^High-quality (hq) sequences only.

^b^A sequence is considered of hq if it is not derived from contaminant species and its vector-trimmed and poor-quality-trimmed PHRED 20 length is >100 bases.

^c^A contig (contiguous sequence) contains two or more ESTs; 3' sequences only.

^d^Number of putative unique transcripts (PUTs) among assembled 3' ESTs equals the number of contigs plus the number of singletons.

^e^Threshold for BLASTX significance versus the non-redundant (NR) database of GenBank is a score value > 50.

### Gene discovery in normalized and non-normalized cDNA libraries

From each of the 20 cDNA libraries, between 1,536 and 24,959 clones were 3'-end sequenced, with the rate of hq sequences ranging from 77.1% to 94.1% and an average EST length of 532 bp to 756 bp in each library (Additional File 1). The rate of gene discovery for each library was assessed from: (1) the number of unique transcripts sequenced from each library; (2) the average number of EST sequences forming contigs; (3) the percentage of ESTs with no similarity to protein sequences in the non-redundant (NR) database of GenBank using BLASTX; (4) the percentage of singleton ESTs; and (5) the percentage of library-specific transcripts. Based on these criteria, all but two of the normalized libraries (i.e., WS-SE-N-A-18 and WS-SE-N-A-19) showed considerably higher rates of gene discovery, and hence higher complexity, than the corresponding non-normalized libraries (Additional File 1). For example, among the six successfully normalized EST libraries, the percentage of unique transcripts identified within the first 1,000 reads averaged 94.7% (92.7% to 95.9%), whereas among the seven corresponding standard EST libraries made from the same RNA samples, the average was only 78.8% (73.8% to 85.6%). The diversity of starting biological materials combined with normalization resulted in low sequence redundancy demonstrated by the presence of only three PUTs (derived from 3'-end ESTs) sequenced in all of the 20 cDNA libraries (Table 3). These three transcripts were identified as translationally controlled tumor protein (209 ESTs), eukaryotic translation initiation factor 5A (115 ESTs) and S-adenosylmethionine synthase (104 ESTs).

**Table 3:** Distribution of ESTs in multiple cDNA libraries

Number of libraries	Number of putative unique transcripts with ESTs in all libraries compared
20	3
19	2
18	2
17	12
16	16
15	22
14	36
13	41
12	66
11	101
10	175

### Quality assessment of FLcDNAs

**Figure 1:**
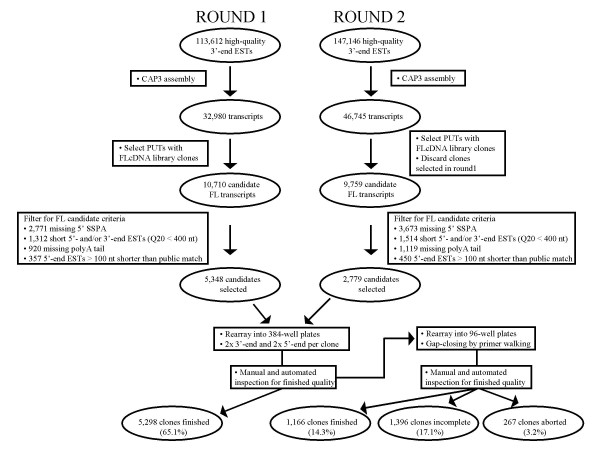
**Clone selection and complete insert sequencing of 6,464 Sitka spruce FLcDNAs**. A total of 20,469 candidate FL transcripts were identified in two consecutive rounds of clone selection involving initially 32,980 and then 46,745 putative unique transcripts (PUTs) derived from a total of 147,146 high-quality 3'-end ESTs. See Methods for complete details of candidate clone selection criteria. Among the 8,127 candidates selected for complete insert sequencing, 5,298 were finished by end reads only, and another 1,166 were finished by end reads plus gap closing using primer walking, yielding a total of 6,464 sequence-verified finished FLcDNAs. An additional 1,396 clones (17.1%) from the starting set of 8,127 will be finished in future work. Only 267 clones (3.2%) were aborted, which supports the success of our strategy for FLcDNA clone selection.

FLcDNAs are defined as individual cDNA clones that contain the complete ORF coding sequence as well as at least partial 5' and 3' UTRs for a given transcript. We prepared three FLcDNA libraries using the biotinylated cap trapper method [45]. All FLcDNA libraries were made from insect-induced tissues of a single Sitka spruce genotype (Table 1). From these libraries, we identified 8,127 cDNA candidate clones for complete insert sequencing, which resulted in 6,464 hq sequence-verified FLcDNA clones (Additional File 2). Analysis of the 6,464 FLcDNA sequences using the CAP3 program ([44]; assembly criteria: 95% identity, 40 bp window) identified 5,197 FLcDNAs as singletons, with the remaining 1,267 grouping into 521 contigs, suggesting a total of 5,718 PUTs represented with finished FLcDNA sequences. The high rate (88.5%) of unique transcript discovery resulted from a successful strategy for selection of a low-redundancy FLcDNA clone set prior to sequence finishing (Figure 1).

**Figure 2:**
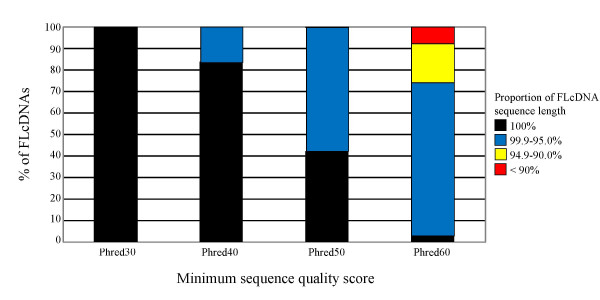
**Validation of sequence quality of FLcDNAs**. Sequence accuracy was measured as the percentage of the 6,464 FLcDNAs which, with 100%, 95.0–99.9%, 90.0–94.9% or <90.0% of their sequence length, exceeded Phred30, Phred40, Phred50 or Phred60 sequence quality thresholds. All 6,464 FLcDNAs exceeded the Phred30 quality thresholds (less than 1 error in 10^3 ^sequenced nucleotides) over 100% of their sequence length. Even at the threshold level of Phred60 (less than 1 error in 10^6 ^sequenced nucleotides) the majority (74.1%) of the FLcDNA sequences met this very high sequence quality score over > 95.0% of their length.

**Figure 3:**
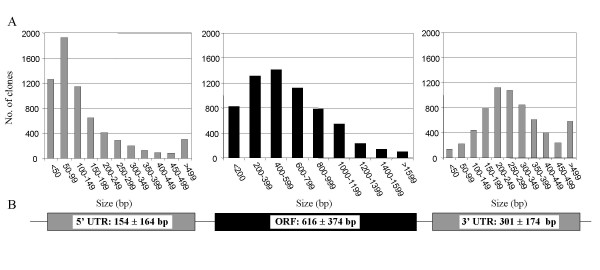
**Distribution of open reading frame (ORF) and 5' and 3' untranslated region (UTR) sizes among the finished 6,464 FLcDNAs (A), and the mean ORF and UTR length (± standard deviation) (B)**. Each finished FLcDNA sequence was examined for the presence of ORFs using the EMBOSS getorf program (version 2.5.0; [69,70]). In each case, the longest stretch of uninterrupted sequence between a start (ATG) and stop codon (TGA, TAG, TAA) in the 5' to 3' direction was taken as the predicted ORF. The presence and coordinates of the 5' second strand primer adaptor sequence (SSPA) and polyA tail were also noted. The regions between the 5'SSPA and the predicted ORF start and between the predicted ORF stop and the polyA tail were taken to be the 5' and 3' UTRs, respectively. The 5' SSPA and 3' polyA tail lengths were not included when determining UTR length.

All 6,464 sequence-verified FLcDNAs achieved a minimum of Phred30 sequence quality at every base (i.e., no more than one error in 10^3 ^bases). The majority were of even higher quality with the minimum and average quality values exceeding Phred45 (less than one error in approximately 3 × 10^4 ^bases) and Phred80 (less than one error in 10^8 ^bases), respectively (Figure 2). We predicted the complete protein-coding ORFs for all 6,464 FLcDNAs (Additional File 2). The average sequenced FLcDNA length (from beginning of the 5' UTR to the end of the polyA tail) was 1,088 ± 404 bp (mean ± SD), and ranged from 401 to 3,003 bp, whereas the average predicted ORF was 616 ± 374 bp and ranged from 30 to 2,583 bp (Figure 3). ORFs could not be detected (i.e., less than 30 bp) for 11 FLcDNAs. The 5' and 3' UTRs averaged 154 ± 164 bp and 301 ± 174 bp, respectively (Figure 3).

**Figure 4:**
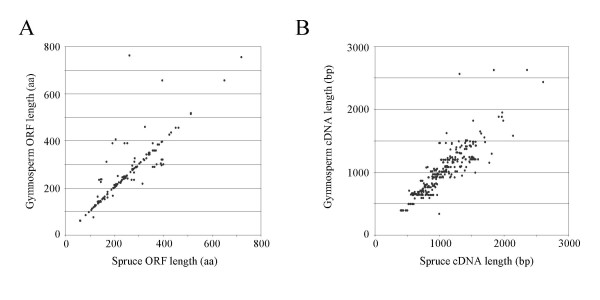
**Validation of spruce FLcDNAs by comparison of ORF lengths (A) and cDNA lengths (B) of 297 spruce FLcDNAs with matching gymnosperm FLcDNAs in the public domain**. The 6,464 FLcDNAs were compared to a collection of 872 gymnosperm sequences from SwissProt using BLASTX ([71]; release 50.1 of June 13^th^, 2006) annotated as full-length (excluding predicted proteins derived from genomic DNA). This comparison identified 297 homologous pairs. A spruce-gymnosperm FLcDNA pair was considered homologous if (1) the best gymnosperm protein BLASTX match exceeded a stringent threshold (% identity ≥ 50%; score value > 95) and (2) the reciprocal TBLASTN analysis identified the same spruce FLcDNA with a score value equal to or within 10% of the best match. ORF and cDNA lengths for gymnosperm sequences were extracted from the SwissProt records, and spruce ORF lengths were predicted using the EMBOSS getorf program. Strong correlations were observed for both ORF and cDNA lengths between spruce and gymnosperm sequences for the available test set of 297 homologous pairs.

To further assess the quality of the FLcDNAs, we performed reciprocal BLAST analysis using 872 known FL sequences from other conifer and gymnosperm species identified in previous entries in the NR database of GenBank. Using a stringent similarity threshold [identity ≥ 50%; BLASTX score value ≥ 95, where alignment scores are calculated based on match, mismatch and gaps in alignments using the default BLAST scoring matrices and parameters] we identified 297 pairs of Sitka spruce and other gymnosperm FLcDNAs. Of these pairs, 244 (82.1%) agreed well with regard to their ORF lengths (Figure 4) and positions of their starting methionine and stop codons (± ten amino acids). For the remaining pairs, the predicted 5' and/or 3' ORF ends did not match, suggesting alternative start or stop codons, splice variants, or the possibility that one of the pair members was truncated or had an incorrectly predicted ORF. Despite the relatively small number of other gymnosperm FL sequences available for pairwise comparison, the high sequence similarity within this dataset indicates that most of the 6,464 FLcDNAs represent true FL transcripts with complete ORFs and correctly annotated start and stop codons.

### Most spruce ESTs have low similarity with angiosperm sequences

**Figure 5:**
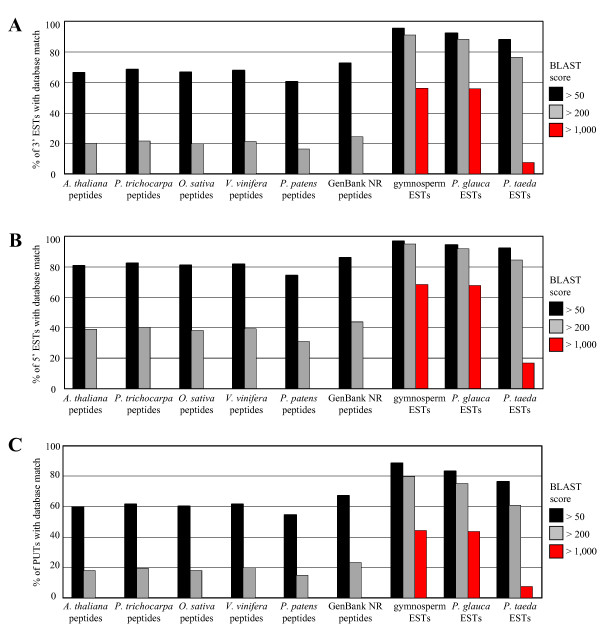
**Sequence annotation of 3' and 5' ESTs and putative unique transcripts (PUTs) against published databases**. Panels A, B and C show the percentage of 3' ESTs, 5' ESTs and PUTs (derived from 3'-end ESTs), respectively, with sequence similarity to entries in nine databases including BLASTX searches against peptides from five sequenced plant genomes (i.e., *Arabidopsis thaliana*, *Populus trichocarpa*, *Oryza sativa*, *Vitis vinifera*, and *Physcomitrella patens*), and all peptides in the non-redundant (NR) database of GenBank; as well as BLASTN searches against 1) all gymnosperm ESTs in dbEST database of GenBank, 2) all *Picea glauca *ESTs in dbEST, and 3) all *Pinus taeda *ESTs in dbEST. Matches were identified using low (score > 50) medium (score > 200) or high (score > 1,000) BLAST stringency thresholds.

Since conifers and other gymnosperms are difficult experimental systems with few functionally characterized proteins, *in silico *annotation of spruce ESTs was performed against predicted peptides from sequenced genomes of four angiosperms (Arabidopsis, rice, poplar, and grapevine) and the moss *Physcomitrella patens*, together with all protein sequences in the NR database of GenBank. Among hq 3'-end ESTs > 400 bases in length (N = 133,065), between 60.5% and 68.6% have matches against each of the five plant genomes with a low stringency BLASTX score of > 50 (Figure 5A and Additional File 3). Using a more stringent threshold of score > 200, between 16.1% and 21.4% of spruce 3'-end ESTs match peptides from each of the five plant genomes of this comparison. BLASTX matches with hq 3'-end ESTs were slightly higher (72.8% and 24.5% at score > 50 and > 200, respectively) when compared to the more comprehensive collection of proteins in the NR database (Figure 5A and Additional File 3). Similar results were obtained using the assembled contig set of 46,745 spruce PUTs derived from 3'-end ESTs (Figure 5C and Additional File 5). Among hq 5'-end ESTs > 400 bases in length (N = 36,505), sequence similarity with proteins predicted from the five plant genome sequences was higher compared to 3' ESTs and PUTs, with between 74.3% and 82.6% (low stringency) and 30.7% and 40.2% (high stringency) of 5'-end ESTs matching each of the plant genomes (Figure 5B and Additional File 4). As observed with 3'-end ESTs and PUTs, an even higher proportion of 5'-end ESTs had BLASTX matches against the NR database (85.9% and 43.8% at score > 50 and > 200, respectively). These results illustrate the challenge of *in silico *annotation of conifer ESTs, even with hq sequences averaging > 650 bases in length.

We also compared the spruce ESTs and PUTs against ESTs from all gymnosperm species combined (dbEST database of GenBank, excluding ESTs reported in this study) using BLASTN. As expected, sequence similarity between the spruce ESTs and published gymnosperm ESTs was high (Figure 5 and Additional Files 3, 4, 5). Among PUTs (derived from 3'-end ESTs), hq 3'-end and 5'-end ESTs > 400 bases in length, 88.6%, 95.4% and 96.9%, respectively, have matches with scores > 50. At higher BLASTN stringency levels (i.e., scores > 200 and > 1,000), sequence matches for PUTs, 3'-end and 5'-end ESTs remain consistently high. Among those PUTs, 3'-end and 5'-end ESTs > 400 bases in length and with no obvious similarity to proteins from the five sequenced plant genomes (at score ≤ 50), 60.0%, 79.1%, and 82.0%, respectively, have BLASTN scores > 200 versus published gymnosperm ESTs (Additional Files 3, 4, 5). When the spruce ESTs are compared against published ESTs from white spruce and loblolly pine, the two gymnosperm species with the most substantial EST collections, a higher proportion of PUTs, and 3'-end and 5'-end ESTs show sequence similarity to white spruce compared to loblolly pine, especially at the highest BLASTN threshold (Figure 5 and Additional Files 3, 4, 5).

### Utility of spruce FLcDNAs for comparative sequence annotation

**Figure 6:**
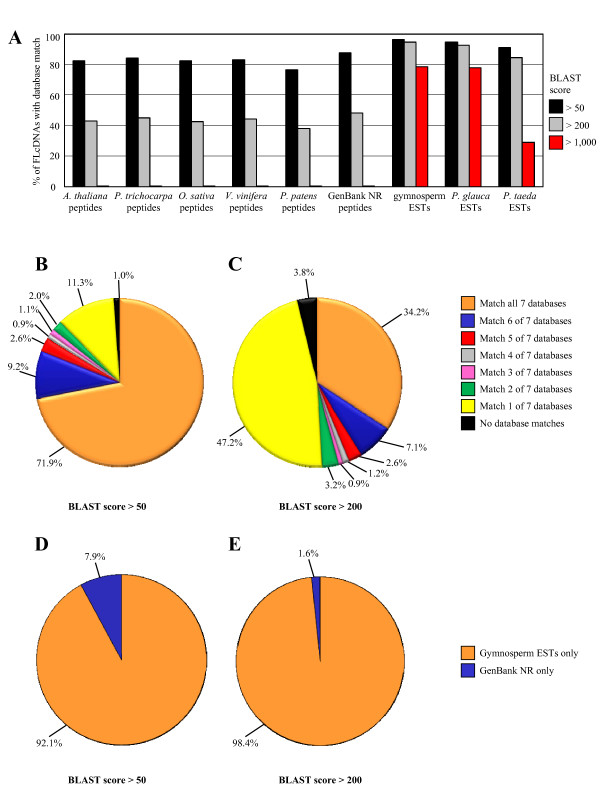
**Sequence annotation of 6,464 high-quality spruce FLcDNAs against published databases**. Panel A shows the percentage of FLcDNAs with sequence similarity to entries in nine databases including BLASTX searches against peptides from five sequenced plant genomes (i.e., *Arabidopsis thaliana*, *Populus trichocarpa*, *Oryza sativa*, *Vitis vinifera*, and *Physcomitrella patens*), and all peptides in the non-redundant (NR) database of GenBank; as well as BLASTN searches against 1) all gymnosperm ESTs in dbEST database of GenBank, 2) all *Picea glauca *ESTs in dbEST, and 3) all *Pinus taeda *ESTs in dbEST. Matches were identified using low (score > 50) medium (score > 200) or high (score > 1,000) BLAST stringency thresholds. Panels B and C show the non-overlapping distribution of matches of spruce FLcDNAs against seven databases (peptides from *A. thaliana*, *P. trichocarpa*, *O. sativa*, *V. vinifera*, *P. patens*, and the NR database of GenBank; and gymnosperm ESTs) at BLAST score thresholds of > 50 and > 200, respectively. Panels D and E show the database source in cases where spruce FLcDNAs matched only a single database in panels C and D at BLAST score thresholds of > 50 and > 200, respectively.

As might be expected, sequence similarity between the 6,464 Sitka spruce FLcDNAs and other gymnosperm ESTs is very high, with 96.5%, 94.6% and 78.7% of FLcDNAs matching published gymnosperm ESTs at low, medium, and high sequence similarity thresholds, respectively (Figure 6A and Additional File 2). As observed with spruce ESTs, sequence similarity was highest between spruce FLcDNAs and white spruce ESTs, with lower similarity observed with loblolly pine ESTs (Figure 6A). Next, the spruce FLcDNAs were compared against predicted proteins from five plant genome sequences and protein sequences in the complete NR database of GenBank. At a low sequence similarity threshold of score > 50, between 76.5% and 84.2% of FLcDNAs matched proteins from each of the plant genomes of this comparison, whereas at a higher threshold of score > 200 the percentages of FLcDNAs with matches in the plant genome sequences ranged from 38.1% to 44.9% (Figure 6A and Additional File 2). Overall, the Sitka spruce FLcDNAs show greater similarity to predicted proteins from sequenced plant genomes compared to the spruce ESTs. The proportion of spruce FLcDNAs with similarity to proteins in the NR database was also higher than spruce ESTs at 87.7% and 47.9% at score > 50 and score > 200, respectively (Figure 6A and Additional File 2).

These results show that FLcDNAs provide a clear advantage over ESTs for large scale *in silico *annotation of spruce sequences. Nevertheless, when using high stringency criteria relevant for *in silico *functional annotation (score values > 200), the comparison of spruce FLcDNAs against the five plant genomes, as well as all plant species in the NR database, still identifies a substantial number of sequences that only show significant matches with other gymnosperms, as opposed to angiosperms. Among the 6,464 spruce FLcDNAs, we found 927 (14.3%) without a reliable match to angiosperm sequences at a low stringency (i.e., BLASTX score ≤ 50), of which 743 (80.1%) match with high sequence similarity (i.e., BLASTN score > 200) to a published gymnosperm EST sequence (Additional File 2). A very small number of spruce FLcDNAs lack sequence similarity to angiosperm or gymnosperm sequences (at score ≤ 50) and display a best match with non-plant species in the NR database of GenBank; 1.0% at score > 50 and 0.3% at score > 200 (Additional File 2). In these cases, the best match is often an insect sequence suggesting small amounts of contaminants in the cDNA libraries.

Comparing the entire spruce FLcDNA dataset against sequences from all species identified that 71.9% (at score > 50) or 34.2% (at score > 200) have matches in all seven datasets (i.e., five plant genomes, the NR database of GenBank, and gymnosperm ESTs) (Figure 6B and 6C). It is notable that at the higher threshold of score > 200, 47.2% of spruce FLcDNAs match only to a single database, and in the vast majority of cases this is a gymnosperm sequence (Figure 6E). Another 1.0% (at score ≤ 50) or 3.8% (at score ≤ 200) of spruce FLcDNA sequences do not align to any sequences in available databases. These sequences could represent genes from spruce (or genes from other contaminant organisms) that have not been sequenced before in any source.

## Discussion

### Spruce ESTs and FLcDNAs enhance conifer genomics resources

Genomics research on conifers has been limited by the lack of a relevant gymnosperm reference genome sequence. The very large size of conifer genomes (10 to 40 Gb; [1]), dominated by repetitive DNA, has been a roadblock to a conifer genome sequence project. Furthermore, the phylogenetic distance between conifers and the well-studied angiosperms is more than 300 million years [2], limiting the utility of angiosperm genome information for research in conifers. To overcome these obstacles to conifer genome research, we have developed two new valuable components for the "conifer genomics toolbox".

First, we have assembled a large collection of high-quality, sequence-verified FLcDNA clones from Sitka spruce, along with a corresponding database of *in silico *annotations (Additional File 2). These FLcDNAs are of very low redundancy. They represent the third largest sequence-verified FLcDNA resource for any plant species, behind only rice [7] and Arabidopsis [4], and are the only substantial FLcDNA resource for a conifer or any other gymnosperm.

Second, we have added a large number of new EST sequences to the public spruce EST collection in GenBank, along with corresponding databases of *in silico *annotations (Additional Files 3, 4, 5). This resource, which was developed from Sitka, white and interior spruce (interior spruce has varying degrees of admixture between white and Engelmann spruce), substantially improves the size and quality of the previously described spruce EST collections [20-23]. The spruce EST collection, along with the ESTs from loblolly pine [15-18], is now one of the two largest EST resources for any conifer species. To enhance gene discovery, we strategically employed library normalization, which had previously not been applied to a conifer EST program. Also, we have added sequences from an until now poorly represented class of tissues representing a biologically important component of conifer defense: insect-, wound- or elicitor-induced tissues.

We identified 46,745 PUTs (19,941 contigs, 26,804 singletons; derived from 3'-end ESTs) in the three species groups surveyed here; Sitka spruce, white spruce, and interior spruce. The rates of PUT discovery for all species combined (31.8%), white spruce only (33.0%) and Sitka spruce only (36.6%) are comparable, as are the ratios of singletons to contigs in each collection. Among contigs from the combined analysis of white and Sitka spruce ESTs, 26.7% contained ESTs from both species, suggesting that ESTs derived from different spruce species representing the same spruce gene often cluster together. The PUTs identified here may represent a substantial portion of the expressed gene catalogue for species of spruce, but a complete genome sequence is needed for assessment of true gene numbers in conifers.

The spruce ESTs described here have already provided the foundation for functional and comparative genomics research on conifer defense against insects, adaptation to the environment, somatic embryogenesis and wood formation, via both transcriptome and proteome analyses [21,34,35,42,46]. They have also allowed development of three types of genetic markers: microsatellites [47,48], single nucleotide polymorphisms (SNP) and conserved orthologous sequences (COS) [41]. The FLcDNA sequences enable rigorous large-scale comparisons of evolutionary patterns at large evolutionary scales (K. Ritland et al., manuscript in preparation).

### Utility of spruce FLcDNAs for functional characterization of gene families including nearly identical paralogous genes

Prior to this work, only a few dozen complete spruce protein sequences were available in the SwissProt database, and no substantial FLcDNA resource was available for any gymnosperm. Using FLcDNAs, detailed pathway annotation, gene expression analysis, and biochemical functional characterization of individual genes and gene families are now possible (S.G. Ralph and J. Bohlmann, manuscript in preparation). The Sitka spruce FLcDNAs have already advanced the discovery and the characterization of conifer defense genes [49-53]. Importantly, Sitka spruce FLcDNAs allow for accurate analysis of closely related members of gene families such as cytochrome P450-dependent monooxygenases or terpenoid synthases (TPS) involved in defense against insects or pathogens [40,54]. For example, TPSs represent a gene family containing many pairs or groups of nearly identical paralogous genes each with a potentially different biochemical function [32]. Our recent mutational analysis of two closely related paralogous Norway spruce di-TPS illustrated that a single amino acid mutation in a background of more than 800 amino acids completely alters biochemical product profiles [55]. Similarly, in rice, the functional divergence of two distinct TPS of primary and secondary metabolism was due to a single amino acid substitution [56]. These examples illustrate the utility of true FLcDNAs for discovery of nearly identical paralogous genes and for functional assessment of gene evolution that is now possible in Sitka spruce.

### Utility of FLcDNAs for conifer proteome and genome characterization

Beyond their importance for functional characterization of individual genes and the analysis of gene families, on an even larger scale, FLcDNAs are also superior to ESTs for overall proteome and genome characterization in a conifer. Because the Sitka spruce FLcDNAs allow for a much more reliable prediction of the complete protein-coding ORF than ESTs, they have been invaluable for proteome predictions and practical proteome analyses [35]. In expectation of future efforts to sequence a conifer genome, FLcDNAs and their ORFs will be essential for the development and training of gene prediction software, as has recently been demonstrated for poplar [8,9].

### Spruce FLcDNAs from insect-induced libraries reveal genes not detected in angiosperms

Comparison of Sitka spruce sequences against angiosperm plants suggests that there are likely a substantial number of genes in the collection of 6,464 FLcDNAs that are either absent in other species, or lack significant sequence similarity for unambiguous identification. In earlier work, Kirst et al. [16] suggested that less than 10% of loblolly pine transcripts lack a related gene in Arabidopsis (defined at a BLASTX E value cutoff of 1e^-10 ^or *ca. *score 60). When we analyzed the spruce FLcDNAs, we found that approximately 14% had no similarity to any angiosperm at a BLASTX stringency of score 50 (slightly lower than that applied by Kirst et al. [16]), based on comparisons to four sequenced angiosperm genomes and all angiosperm sequences in the NR database. This slightly higher rate may be the result of sequencing libraries made from tissues induced by insect attack, which may disproportionally represent genes with specialized functions in conifer defense that are subject to high levels of natural selection due to biotic interaction. By contrast, genes involved in xylem development and wood formation appear to be well conserved in angiosperms and conifers [16,46].

## Conclusion

The 206,875 ESTs and 6,464 FLcDNAs and the corresponding *in silico *annotated sequence databases provide a new and valuable genomics resource for species of spruce, as well as for gymnosperms in general. Our emphasis on FLcDNAs and ESTs from cDNA libraries constructed from herbivore-, wound- or elicitor-treated induced spruce tissues, along with incorporating normalization to capture rare transcripts, gives a rich conifer EST resource which also apparently contains a substantial number of transcripts with no obvious sequence similarity to known angiosperm sequences. Recent research has begun to fully realize the application of these EST and FLcDNA sequences, and FLcDNA clones.

## Methods

### cDNA library construction

Details of the isolation of total and poly(A)^+ ^RNA are described in Additional File 6. Standard cDNA libraries were directionally constructed (5' *Eco*RI and 3' *Xho*I) using 5 μg poly(A)^+ ^RNA and the pBluescript II XR cDNA Library Kit, following manufacturer's instructions (Stratagene, La Jolla, USA) with modifications. First-strand synthesis was performed using Superscript II reverse transcriptase (Invitrogen, Carlsbad, USA) and an anchored oligo d(T) primer [5'-(GA)_10_ACTAGTCTCGAG(T)_18_VN-3']. Size fractionation was performed on *Xho*I-digested cDNA prior to ligation into vector using a 1% NuSieve GTG low melting point agarose gel (BioWhittaker Molecular Applications, Walkersville, USA) and β-agarase (New England Biolabs, Ipswich, USA) to isolate cDNAs from 300 bp to 5 kb. Select cDNA libraries were normalized to Cot = 5 using established protocols [13,14]. Library plasmids were propagated in ElectroMAX DH10B T1 Phage Resistant Cells (Invitrogen). FLcDNA libraries were directionally constructed (5' *Xho*I and 3' *BamH*I) according to methods of Carninci and Hayashizaki [57] and Carninci et al. [58], with modifications described in Additional File 6.

### DNA sequencing and sequence filtering

Details of bacterial transformation with plasmids, clone handling, DNA purification and evaluation, and DNA sequencing are provided in Additional File 6. Sequences from each cDNA library were closely monitored to assess library complexity and sequence quality. DNA sequence chromatograms were processed using the PHRED software (versions 0.000925.c and 0.020425.c) [59,60]. Sequences were quality-trimmed according to the high-quality (hq) contiguous region determined by PHRED and vector-trimmed using CROSS_MATCH software [61]. Sequences with less than 100 high quality bases (Phred20 or better) after trimming and sequences with polyA tails of ≥ 100 bases were removed from the analysis. Also removed were sequences representing bacterial, yeast or fungal contaminations identified by sequence alignments using BLAST [62,63] to *E. coli *K12 DNA sequence (GI: 6626251), *Saccharomyces cerevisiae *(GenBank, ), *Aspergillus nidulans *(TIGR ANGI.060302), and *Agrobacterium tumefaciens *(custom database generated using SRS, Lion Biosciences). Sequences were also compared to the NR protein database [67]. Top ranked BLAST matches to species other than plants with score values > 60 were flagged as contaminants and were removed from the EST dataset. EST sequences have been deposited in the dbEST database of GenBank [DR448912 to DR451924; DR463975 to DR595214; CV720218 to CV720219; CO203067 to CO245079; CO250245 to CO252887; CO252989 to CO253183; CO253265 to CO257405; CO257513 to CO258618; CN480886 to CN480910].

### Selection of candidate FLcDNA clones and sequencing strategy

All 3'-end ESTs remaining after filtering were clustered and assembled using CAP3 ([44]; assembly criteria: 95% identity, 40 bp window). The resulting contigs and singletons were defined as the putative unique transcript (PUT) set. PUTs with a cDNA clone from a FLcDNA library were selected as candidates for complete insert sequencing. Candidate clones from FLcDNA libraries were single-pass sequenced from both 3'- and 5'-ends, and both sequences were used for subsequent clone selection. Clones were screened for the presence of a polyA tail (3'-end EST) and the second-strand primer adaptor (SSPA; 5'-ACTAGTTTAATTAAATTAATCCCCCCCCCCC-3'; 5'-end EST). Clones lacking either of these features were eliminated. A polyA tail was defined as at least 12 consecutive, or 14 of 15 "A" residues within the first 30 bases of the 3'-end EST (5' to 3'). The presence of the SSPA was detected using the Needleman-Wunsch algorithm limiting the search to the first 30 bases of the 5'end EST (5' to 3'). The SSPA was defined as eight consecutive "C" residues and a ≥ 80% match to the remaining sequence (5'-ACTAGTTTAATTAAATTAAT-3'). In each case, the algorithms used to detect the 5' and 3' clone features were set to produce maximal sensitivity while maintaining a 0% false positive rate, as determined using test data sets. Candidate clones for which either of the initial 5'-end or 3'-end EST sequences had a Phred20 quality length of < 400 bases were also excluded. Finally, any clone with a 5'-end EST which had a BLASTN match (score value > 300) to a gymnosperm EST in the public domain (excluding ESTs from this collection) and was > 100 bases shorter at the 5' end than the matching EST was flagged as truncated at the 5' end and was excluded. For each PUT represented by multiple candidate clones after filtering, the clone with the longest 5' sequence was selected for complete insert sequencing. Insert sizing using colony PCR and vector primers was performed on 1,634 cDNA clones with an average insert size of *ca. *1,250 bp. Based on this information, a sequencing strategy emphasizing the use of end reads was chosen. Using end reads only, 5,298 clones were complete insert sequenced to a high quality. Among this set, the average sequenced insert size was 1,005 ± 282 bp (average ± SD) with an average of 5.93 ± 0.51 end reads required to finish. Using a combination of end sequencing and primer walking, an additional 1,166 clones were complete insert sequenced, with an average insert size of 1,653 ± 447 bp, and requiring six end reads and 2.62 ± 1.51 internal primer reads per clone.

### Sequence finishing of FLcDNA clones

FLcDNA clones selected for complete sequence finishing were rearrayed into 384-well plates, followed by two additional rounds of 5'-end and 3'-end sequencing using vector primers. All sequences from an individual clone were then assembled using PHRAP (version 0990329) [59,60]. To meet our hq criteria, the resulting clone consensus sequence was required to achieve a minimum average score of Phred35, with each base position having a minimum score of Phred30. Each base position also required at least two sequences, each with a minimum quality of Phred20, that were in agreement with the consensus sequence (i.e., no high-quality discrepancies). Clones that did not meet these finishing criteria or that had gaps after three rounds of end sequencing were then subjected to successive rounds of sequencing using custom primers designed using the Consed graphical tool version 14 [64] until the required quality levels were achieved. Regardless of the finishing strategy, all clones that did not meet the minimum finishing criteria according to an automated pipeline were manually examined. Clones were aborted if they were manually verified to lack the minimum finishing criteria, did not possess the cloning structures, were identified as chimeric, were refractory to sequence finishing due to the presence of a "hard-stop", or if errors were identified in the re-array of glycerol stocks. FLcDNA sequences have been deposited in GenBank [EF081469 to EF087932].

### Comparative sequence annotation

The following databases were used to perform BLAST analyses for EST and FLcDNA annotation: 1) *Arabidopsis thaliana*, The Arabidopsis Information Resource version 7, release date April 25^th^, 2007, 31,921 peptides [65]; 2) *Populus trichocarpa*, Joint Genomes Institute (JGI) version 1.1, release date September 16^th^, 2006, 45,555 peptides [66]; 3) *Oryza sativa*, National Center for Biotechnology Information (NCBI), download date April 8^th^, 2008, 177,254 peptides [67]; 4) *Vitis vinifera*, NCBI, download date April 8^th^, 2008, 55,851 peptides [67]; 5) *Physcomitrella patens*, JGI version 1.1, release date January 4^th^, 2008, 35,938 peptides [68]; 6) NR database of GenBank, NCBI release 162, release date October 15^th^, 2007, 5,372,238 peptides [67]; 7) gymnosperm ESTs in NCBI (excluding ESTs reported in this study), download date April 8^th^, 2008, 622,923 ESTs [67]; 8) *Picea glauca *ESTs in NCBI (excluding ESTs reported in this study), download date April 8^th^, 2008, 197,042 ESTs [67]; 9) *Pinus taeda *ESTs in NCBI, download date April 8^th^, 2008, 328,628 ESTs [67].

## Authors' contributions

JB and SGR conceived and directed this study. SGR, NK, DC, and CO developed full-length cDNA and EST libraries. SGR, HJEC, RK and JB analyzed data with assistance from the coauthors. RAH, SJMJ and MM directed sequencing and bioinformatics work at the GSC. JB and SGR wrote the paper. All authors read and approved the final manuscript.

## Supplementary Material

Additional File 1 **cDNA library summary statistics.** Sequencing statistics organized by cDNA library source for spruce expressed sequence tags. Click here for file

Additional File 2 **Full-length cDNA inventory.** Predicted protein-coding features, annotation, and GenBank accession numbers for the Sitka spruce full-length cDNA collection. Click here for file

Additional File 3 **3'-end EST inventory.** Detailed annotation and GenBank accession numbers for the complete set of spruce 3'-end ESTs. Click here for file

Additional File 4 **5'-end EST inventory.** Detailed annotation and GenBank accession numbers for the complete set of spruce 5'-end ESTs. Click here for file

Additional File 5 **PUT inventory.** Detailed annotation for the complete set of putative unique transcripts. Click here for file

Additional File 6 **Supplemental methods.** Detailed methods for RNA isolation, full-length cDNA library construction, bacterial transformation with plasmids, clone handling, DNA purification and evaluation, and DNA sequencing. Click here for file
