# Supplementary material for: A conifer genomics resource of 200,000 spruce (Picea spp.) ESTs and 6,464 high-quality, sequence-finished full-length cDNAs for Sitka spruce (Picea sitchensis)
Source: BMC Genomics. 2008 Oct 14;9:484. doi: 10.1186/1471-2164-9-484 (PMC2579922; doi:10.1186/1471-2164-9-484)
Supplement: Additional File 1 — cDNA library summary statistics. Sequencing statistics organized by cDNA library source for spruce expressed sequence tags. [file 1471-2164-9-484-S1.doc]

| cDNA Library | Total no. 3’ ESTs (5' ESTs) | No. and % high-quality ESTs^c^ | Avg. EST length (bp)^d^ | No. and % unique transcripts^e^ | Avg. no. contig members^f^ | % No BLASTX match vs. NR^g^ | % Singleton^h^ | % Library-specific transcripts^i^ |
| --- | --- | --- | --- | --- | --- | --- | --- | --- |
| WS-ES-A-1^a^ | 4,992 | 4,284 (85.8) | 532 | 2,548 (76.8) | 3.92 | 36.8 | 45.6 (65.7) | 1.31 (2.91) |
| WS-PS-A-2^a^ | 1,536 | 1,351 (87.9) | 603 | 1,000 (76.1) | 3.28 | 27.7 | 62.6 (64.9) | 0.36 (0.92) |
| WS-PS-N-A-8^b^ | 18,814 | 16,602 (88.2) | 661 | 10,201 (95.2) | 2.90 | 35.6 | 41.1 (90.9) | 7.78 (11.28) |
| WS-X-A-3^a^ | 3,358 | 2,591 (77.1) | 537 | 1,812 (80.5) | 3.25 | 47.0 | 56.6 (69.5) | 0.80 (1.76) |
| WS-X-N-A-9^b^ | 15,744 | 14,149 (89.8) | 542 | 9,161 (95.9) | 2.73 | 57.9 | 44.4 (92.1) | 7.60 (9.62) |
| IS-B-A-4^a^ | 1,536 | 1,359 (88.4) | 605 | 1,040 (80.6) | 2.90 | 30.6 | 64.2 (69.3) | 0.44 (0.92) |
| IS-B-A-7^a^ | 1,536 | 1,379 (89.7) | 589 | 949 (74.7) | 3.56 | 26.7 | 56.6 (62.5) | 0.39 (0.94) |
| IS-B-N-A-10^b^ | 24,959(3,072) | 22,561 (90.3) | 688 | 13,380 (94.7) | 3.01 | 34.6 | 39.1 (90.5) | 12.66 (15.33) |
| SS-R-A-5^a^ | 3,072 | 2,713 (88.3) | 546 | 1,986 (85.6) | 3.31 | 43.8 | 61.6 (76.7) | 1.25 (1.84) |
| SS-R-N-A-11^b^ | 17,664 | 15,512 (87.8) | 539 | 10,958 (95.9) | 2.68 | 56.4 | 53.2 (92.2) | 11.18 (10.54) |
| WS-PP-A-6^a^ | 3,072 | 2,827 (92.0) | 584 | 1,923 (80.6) | 3.15 | 34.0 | 53.1 (69.7) | 0.65 (1.92) |
| WS-PP-N-A-12^b^ | 12,288(3,072) | 11,574 (94.1) | 663 | 6,899 (92.7) | 3.06 | 35.5 | 40.0 (86.6) | 3.76 (7.87) |
| SS-IB-A-FL-13^a^ | 6,912(6,912) | 5,626 (81.3) | 707 | 3,009 (76.9) | 3.93 | 15.6 | 37.6 (66.7) | 1.33 (3.82) |
| SS-IL-A-FL-14^a^ | 19,968(19,968) | 17,666 (88.4) | 752 | 7,281 (82.9) | 4.72 | 13.7 | 25.4 (72.9) | 5.19 (12.01) |
| SS-IB-A-FL-15^a^ | 8,448(8,448) | 7,681 (90.9) | 732 | 3,402 (76.4) | 4.36 | 17.8 | 27.7 (64.1) | 1.64 (5.22) |
| WS-SE-A-16^a^ | 1,536 | 1,327 (86.3) | 756 | 928 (74.0) | 3.46 | 17.2 | 57.7 (62.6) | 0.42 (0.90) |
| WS-SE-N-A-18^b^ | 3,840 | 3,530 (91.9) | 736 | 1,541 (70.6) | 3.65 | 24.3 | 22.4 (51.9) | 0.73 (2.40) |
| WS-SE-N-A-19^b^ | 3,840 | 3,359 (87.4) | 709 | 1,456 (68.4) | 3.59 | 29.2 | 21.5 (49.8) | 0.70 (2.28) |
| WS-MC-A-17^a^ | 1,536 | 1,298 (84.5) | 749 | 917 (73.8) | 4.15 | 19.6 | 61.3 (65.5) | 0.51 (0.88) |
| WS-MC-N-A-20^b^ | 10,752 | 9,757 (90.7) | 742 | 6,313 (93.8) | 2.95 | 29.2 | 46.6 (88.1) | 5.41 (6.63) |
| ^a^Standard or full-length cDNA library (white background).  ^b^Normalized cDNA library (gray background).  ^c^A sequence is considered high-quality (hq) if its trimmed PHRED 20 length is >100 bases after vector only, low-quality and contaminating yeast, bacterial or fungal sequences are removed; 3’ sequences only. The number in parentheses is the number of hq transcripts as a percentage of total sequences.  ^d^Hq 3’ sequences only.  ^e^Number of putative unique transcripts among assembled hq ESTs equals the number of contigs plus the number of singletons. The number in parentheses is the percentage of unique transcripts among the first 1,000 hq sequences assembled.  ^f^Average number of contig members where a contig (contiguous sequence) contains two or more hq ESTs.  ^g^Percentage of putative unique transcripts with no significant BLASTX similarity versus the non-redundant (NR) database of GenBank (score value > 50).  ^h^Percentage singletons is the number of singletons in a library divided by the total number of hq sequences. The number in parentheses is the percentage of singletons among the first 1,000 hq sequences assembled.  ^i^Percentage of library-specific unique transcripts calculated by adding the number of contigs and singletons that were present only in a single cDNA library divided by the total number of putative unique transcripts in the hq EST collection (46,745). The number in parentheses is the percentage of all hq sequences (147,146) derived from a given library. | | | | | | | | |

**Additional file 1.** **Spruce cDNA library summary statistics**
