# Supplementary material for: A conifer genomics resource of 200,000 spruce (Picea spp.) ESTs and 6,464 high-quality, sequence-finished full-length cDNAs for Sitka spruce (Picea sitchensis)
Source: BMC Genomics. 2008 Oct 14;9:484. doi: 10.1186/1471-2164-9-484 (PMC2579922; doi:10.1186/1471-2164-9-484)
Supplement: Additional File 6 — Supplemental methods. Detailed methods for RNA isolation, full-length cDNA library construction, bacterial transformation with plasmids, clone handling, DNA purification and evaluation, and DNA sequencing. [file 1471-2164-9-484-S6.doc]

**Supplemental methods**

**Reagents**

Unless otherwise mentioned, all other reagents and solvents were from Fischer Scientific (Pittsburgh, USA), Sigma-Aldrich (St. Louis, USA), EM Science (Darmstadt, Germany) or Invitrogen (Carlsbad, USA).

**RNA isolation**

Total RNA was isolated from all tissues, except somatic embryos, according to the protocol of Kolosova et al. [1]. Total RNA from somatic embryos was isolated using the RNAqueous Midi Kit (Ambion, Austin, USA). Total RNA was quantified and quality checked by spectrophotometer and agarose gel. Poly(A)^+^ RNA was isolated by oligo d(T) cellulose using the Poly(A) Pure kit (Ambion), following manufacturer’s instructions. RNA was also evaluated for integrity and the presence of contaminants using reverse transcription with Superscript II reverse transcriptase (Invitrogen) with an oligo d(T_18_) primer and αP^32^ dGTP incorporation. After removal of unincorporated nucleotides using gel filtration columns (Microspin S-300 HR columns, Amersham Pharmacia Biotech, Buckinghamshire, UK), the resulting cDNA smear was resolved using a vertical 1% agarose alkaline gel and visualized using a Storm 860 phosphorimager (Amersham Pharmacia Biotech).

**Full-length cDNA library construction**

First-strand synthesis was performed using 20 µg of heat-denatured and methyl mercury-treated poly(A)^+^ RNA combined with a first-strand primer containing *Bam*HI and *Sst*I restriction sites [5`-(GA)_5_AAGGATCCAAGAGCTC(T)_16_VN-3`]. In order to monitor library production, the first-strand synthesis master mix was divided into 75 μL and 25 μL aliquots, with 10 μL of αP^32^ dGTP added as tracer to the smaller aliquot. RNA and primer were combined in 10 μL, incubated at 65˚C for 5 min, and cooled to 4˚C. To minimize interfering RNA secondary structures, 1 μL of 100 mM methyl mercury was added to the RNA-primer mixture, incubated for 1 min at room temperature (RT), followed by addition of 2 μL of 700 μM ß-mercaptoethanol and a second 5 min incubation at RT. For reverse transcription, the RNA-primer mixture was added to a mixture containing 5% glycerol, 1X first-strand buffer (Invitrogen), 0.01 M dithiothreitol, 500 μM (each) dTTP, dGTP, dATP and 5-methyl-dCTP, 0.55 M saturated trehelose, 0.05 μg/μL bovine serum albumin, 1 μL RNase Out (40 U/μL; Invitrogen) and 10 μL Superscript II reverse transcriptase (200 U/μL; Invitrogen) in a final volume of 100 μL. Reverse transcription was performed as follows: step 1, 45˚C for 2 min; step 2, gradient annealing: cool to 35˚C over 1 min; step 3, complete annealing: 35˚C for 2 min; step 4, 45˚C for 5 min; step 5, positive ramp to 60˚C at 0.1˚C/s; step 6, 55˚C for 2 min; step 7, 60˚C for 2 min; then repeat steps 6-7 ten times; followed by a 4˚C hold. Incorporation of radioactivity was used to estimate the yield of cDNA as described in Carninci and Hayashizaki [2]. The cDNA was then treated with proteinase K, phenol/chloroform- and chloroform-extracted, and ethanol precipitated as described previously [2].

Prior to biotinylation, the diol group of the cap and 3’-end of RNA was oxidized in a final volume of 50 μL containing 44 μL of resuspended cDNA, 66 mM sodium acetate (pH 4.5) and 5 mM NaIO_4_, then incubated at 4˚C in the dark for 45 min, followed by the addition of 0.5 μL 10% SDS, 5 μL 5 M NaCl and 50 μL 100% isopropanol. The pellet was precipitated by centrifugation, washed twice with 70% ethanol, and then resuspended in 50 μL H_2_O. The cap was biotinylated in a final volume of 210 μL by adding 5 μL 1 M sodium acetate buffer (pH 6.1), 5 μL 10% SDS and 150 μL 10 mM biotin hydrazide long arm (Vector Biosystem) for 10-16 hrs at RT. The cDNA was then precipitated, washed and resuspended in 70 μL 0.1X TE as described in Carninci et al. [3].

To capture FLcDNAs, 500 μL MPG-streptavidin beads (Millipore, Bellerica, USA) were incubated with 100 μg of DNA-free yeast tRNA (50 μg/μL) at 4˚C for 30 min. Beads were separated with a magnetic stand (Promega, Madison, USA) and washed three times with 500 μL of a washing/binding solution [2 M NaCl, 50 mM EDTA (pH 8.0)]. To 70 μL of cDNA we added 20 μL RNase I 10x buffer (Promega) and 10 μL RNase I and incubated at 37˚C for 15 min. The reaction was terminated by adding 100 μg tRNA and 100 μL of 5 M NaCl. Biotinylated and RNase-treated cDNA were combined with washed beads and gently rotated for 30 min at RT. Beads were then separated on a magnetic stand and washed to remove non-specifically bound cDNAs as described in Carninci et al. [3]. Captured FLcDNAs were released from beads by adding 50 μL of a 50 mM NaOH and 5 mM EDTA solution and incubating for 10 min at RT. Beads were separated by magnet, and the eluted cDNA transferred to a tube containing 50 μL 1 M Tris-HCl (pH 7.5) and stored at 4˚C. The elution cycle was repeated until 80% of cDNA was recovered (as measured by radioactivity with a Geiger counter). The cDNA was then treated with proteinase K, phenol/chloroform- and chloroform-extracted, and ethanol precipitated as described in Carninci and Hayashizaki [2], and resuspended in 50 μL 0.1X TE. Short nucleotide products (< 400 bp) were then removed using a Microspin S-400 HR column (Amersham Pharmacia Biotech) according to manufacturer instructions, followed by precipitation of the recovered cDNA, two washes with 70% ethanol, and resuspension in 33 μL of H_2_O.

Oligo-dG tailing was performed in a final volume of 50 μL containing heat-denatured cDNA sample, 10 μL of 5x TdT buffer, 5 μM dGTP and 2 μL of terminal deoxytransferase (15 U/μL). Samples were incubated at 37˚C for 30 min, with the reaction terminated by adding 1 μL of 0.5M EDTA, followed by proteinase K digestion, phenol/chloroform and chloroform extraction, ethanol precipitation, and resuspension in 39 μL H_2_O [2]. Tail length was checked as described by Carninci and Hayashizaki [2]. Second strand synthesis was performed using the oligo-dG-tailed cDNA, 6 μL of a 100 ng/μL *Xho*I-containing primer [5’-(GA)_7_TTCTCGAGTTAATTAAATTAAT(C)_13_-3’], 6 μL ExTaq buffer (Takara, Otsu, Japan), 6 μL of 2.5 mM (each) dNTPs and 3 μL ExTaq polymerase (5 U/μL; Takara) in a 60 μL volume. Ten μL of the master mix was then transferred to a second tube containing 0.5 μL of αP^32^ dGTP and processed identically in order to estimate the second-strand yield [2]. PCR conditions were: step 1, 55˚C for 5 min; step 2, negative ramp to 35˚C at 0.3˚C/min; step 3, 35˚C for 10 min; step 4, 68˚C for 20 min then repeat from step 3; and a final 4˚C hold. The second-strand cDNA was then proteinase K treated, phenol/chloroform- and chloroform-extracted, ethanol precipitated, and resuspended in 44 μL H_2_O [2], followed by *Bam*HI and *Xho*I digestion using 25 U of each enzyme per 1 μg cDNA. Following another round of proteinase K digestion, phenol/chloroform and chloroform extraction, and ethanol precipitation, the cDNA was purified with a CL-4B spin column (Amersham Pharmacia Biotech) according to manufacturer’s instructions. The purified cDNA was then ligated into *Bam*HI- and *Xho*I-digested pBluescript II SK+ vector using standard procedures. Plasmid transformation, library amplification and final plasmid purification were performed according to procedures for the pBluescript II XR cDNA library construction kit (Stratagene, La Jolla, USA).

The average insert size of all cDNA libraries was routinely determined by performing colony PCR on 48 randomly selected bacterial colonies from the amplified library using –21 M13 forward (5’-TGTAAAACGACGGCCAGT-3’) and M13 reverse (5’-CAGGAAACAGCTATGAC-3’) primers. PCR amplicons were resolved on 1% agarose gels and visually compared to DNA size markers λ HindIII and 1 kb ladder (Invitrogen).

**Transformation and colony picking**

A 1 µL aliquot of ligation mix from each cDNA library was transformed by electroporation into 40 µL of *E. coli* DH10B T1 resistant cells (Invitrogen). Transformed cells were recovered using 1 mL of SOC medium (Invitrogen) and plated onto 22 cm x 22 cm agar plates (Genetix, Newmilton, UK) containing 2xYT agar and 100 µg/µL Ampicillin. Agar plates were incubated overnight at 37ºC for 16 h. Bacterial colonies were picked from the agar plates and arrayed into 384-well microtiter plates (Genetix) containing 60 µL of 2xYT medium plus 7.5% glycerol (made in-house) using the Genetix Q-Pix automated colony picker (Genetix). Plates were incubated at 37ºC for 18 h then each microtiter plate was inspected for wells that contained no growth.

## Rearray of glycerol stock clones

## Clones were rearrayed using a Genetix Q-Pix instrument. Clones were selected from originally sequenced clones and inoculated into 384-well microtiter plates (Genetix) containing 60 µL of 2xYT medium plus 7.5% glycerol (made in-house) and 100 µg/µL Ampicillin. The newly created stock plates were incubated at 37ºC for 18 h then each microtiter plate was inspected for wells that contained no growth. If any wells had no growth, the stock plates were stamped into a new 384-well microtiter plates (Genetix), and the missing clones were inoculated by hand to ensure a complete set of clones before proceeding.

**Culturing and DNA purification of plasmid clones**

Two microliters of bacterial culture was transferred from the 384-well microtiter plate into a 240 µL 384-deep well diamond plate (Axygen, Union City, USA) containing 60 µL of 2xYT medium and 100 µg/mL Ampicillin or a 96-well Beckman plate (Beckman Coulter, Fullerton, USA) containing 1200 µL 2xYT and 100 µg/mL Ampicillin using a 384-well or 96-well slotted inoculator (V&P Scientific, San Diego, USA). Inoculated plates were sealed with AirPore^TM^ tape (Qiagen, Hilden, Germany) and placed into a 37ºC shaking incubator (New Brunswick Scientific C25 Incubator Shaker, Edison, USA) at 350 rpm for 18 h (384-well) or 20 h (96-well). After the incubation period, cultures were removed and each plate inspected for growth and contamination; all “no-grows” and observations were recorded. 96-well cultures were centrifuged to pellet cells, the media was decanted, and pellets were dried and frozen until ready for use. Plasmid DNA was isolated from cultures using alkaline lysis with the following modifications that have been implemented for the standard Genome Sciences Centre template production pipeline (384-well). Culture blocks from both replicates were removed from the 4ºC refrigerator and vortexed using a multi-tube floor vortexer (VWR, West Chester, USA) for 5 min at maximum speed (or until all cells appeared resuspended). A Titertek MapC2 (Titertek, Huntsville, USA) liquid handling device was used to dispense 60 µL of Lysis Buffer (Qiagen Buffer P2). After 5 min of lysis, 60 µL of Neutralization Buffer (Qiagen Buffer P3) was added. **Plates were tape sealed (Edge Biosystems clear tape, Gaithersburg, USA**) and vortexed on a multi-tube vortexer at maximum speed for 2 min prior to centrifugation at 4250 x g for 45 min in a Jouan KR422 centrifuge. Lysate (120 µL) were transferred from pelleted culture blocks into daughter 240 µL 384-deep well diamond plates containing 90 µL per well 100% isopropanol using a 384-well hydra (Robbins Scientific, Sunnyvale, USA**). Destination plates were sealed (Edge biosystems clear tape**) and mixed by inversion, followed by centrifugation at 2830 x g for 15 min in an Eppendorf 5810R centrifuge (Brinkmann Instruments, Westbury, USA). After centrifugation, the isopropanol was decanted, the DNA pellet washed with 50 µL 80% ethanol using a Robbins 384-well Hydra, and the plates left to dry upright for 3 h on the benchtop. DNA pellets were resuspended in 10 mM Tris-HCl pH 8.0 (in-house) containing 10 µg/mL RNase A (Qiagen) and vortexed for 1 min at maximum speed on a multi-tube vortexer. Plates were briefly centrifuged at low speed before storage at 4ºC overnight then transferred to a -20ºC freezer until required for DNA evaluation and sequencing reactions.

**DNA evaluation**

DNA preparations were evaluated by agarose gel electrophoresis. A 1.5 µL aliquot of prepared and purified total DNA was combined with 1.5 µL of 5x Bromophenol blue loading buffer (0.21% Bromophenol blue; 12.5% ficoll) and 2 µL was loaded onto a 1xTAE (Tris/Acetate/EDTA) 1.2% agarose gel. Samples were loaded using a 12-channel loader (Hamilton, Reno, USA) along with 3 ng of 1 kb plus DNA marker (Invitrogen). Gels were run at 120 volts for 90 min in 1xTAE buffer followed by staining for 35 min in 1x SYBR Green Nucleic Acid stain (Cambrex, Baltimore, USA). Gels were scanned using a Fluorimager 595 (Amersham Pharmacia Biosciences) scanner. The image was visually examined for genomic DNA, presence and quality of DNA, as well as contamination.

**DNA sequencing**

DNA sequencing reactions were set up in 384-well clear optical reaction plates (Applied Biosystems, Foster City, USA) using the Biomek FX workstation (Beckman Coulter) for liquid transfers. In each 5 µL total volume reaction the following were added: 3 µL of purified plasmid DNA (~45 ng/µL), 0.26 µL of sequencing primer (5 pmol/µL, Invitrogen), 0.43 µL of 5x reaction buffer (Applied Biosystems Big Dye Terminator 5X Sequencing Buffer), 0.77 µL of Ultrapure water (Invitrogen), and 0.54 µL of BigDye v.3.1 ready reaction mix (Applied Biosystems). Sequence data were obtained using -21M13 forward (5'-TGTAAAACGACGGCCAGT-3'), and M13 reverse (5'-CAGGAAACAGCTATGAC-3') or custom primers on each set of replicate plates. Thermal cycling were performed on PTC-225 thermal cyclers (Bio-Rad, Hercules, USA) with parameters of 35 cycles at 96°C for 10 s, 52°C for 5 s using -21M13 forward primer [or 43°C for M13 reverse], 60°C for 3 min, followed by incubation at 4°C. Reaction products were precipitated by adding 2 µL of 125 mM EDTA (pH 8.0) and 18 µL of 95% ethanol per well followed by centrifugation at 2750 x g for 30 min in an Eppendorf 5810R centrifuge. The EDTA/ethanol was immediately decanted and reaction products washed with 70% ethanol. The 384-well cycle plates were allowed to dry inverted on bench top for 15 min. Samples were resuspended in 10 µL of Ultrapure water and analyzed using a 3730XL DNA analyzer (Applied Biosystems).

# Supplemental text references

1. Kolosova N, Miller B, Ralph S, Ellis BE, Douglas C, Ritland K, Bohlmann J: **Isolation of high-quality RNA from gymnosperm and angiosperm trees**. *Biotechniques* 2004, **36**: 821-824.

2. Carninci P, Hayashizaki Y**:** **High-efficiency full-length cDNA cloning.** *Methods Enzymol* 1999, **303**: 19-44.

3. Carninci P, Shibata Y, Hayatsu N, Sugahara Y, Shibata K, Itoh M, Konno H, Okazaki Y, Muramatsu M, Hayashizaki Y: **Normalization and subtraction of cap-trapper-selected cDNAs to prepare full-length cDNA libraries for rapid discovery of new genes.**  *Genome Res* 2000, **10**: 1617-1630.
